# The Natsal-SF Measure of Sexual Function: Comparison of Three Scoring Methods

**DOI:** 10.1080/00224499.2014.985813

**Published:** 2015-02-10

**Authors:** Kyle G. Jones, Kirstin R. Mitchell, George B. Ploubidis, Kaye Wellings, Jessica Datta, Anne M. Johnson, Catherine H. Mercer

**Affiliations:** ^a^ Research Department of Infection and Population Health, University College London; ^b^ Department of Social and Environmental Research, London School of Hygiene and Tropical Medicine

## Abstract

The Natsal-SF is a psychometrically validated measure of sexual function for use in community health surveys, derived from 17 questions reflecting three components of sexual function. Scoring requires knowledge of complex statistical modeling and, given the methodological complexities, we assessed the validity of two simplified scoring methods calculated using the factor loadings produced when originally modeling the Natsal-SF items. Method 1 uses these factor loadings to three decimal places, while method 2 assigns whole numbers to each item based on the factor loadings. Scores from these simplified methods are compared to the original score using correlation coefficients, by comparing the distributions and the scores of each method in a linear regression model with key variables. We found scores from the simplified methods both correlate highly with the original score, and the distributions of scores closely match. The simplified methods result in different regression coefficients for gender and relationship context but estimate the coefficients of all other variables similarly to the original method. While the Natsal-SF should ideally be scored using latent variable modeling, the simplified methods perform well so can be used in similar contexts, increasing the utility of the Natsal-SF and enabling future studies to measure sexual function more comprehensively.

Quality-of-life surveys and epidemiological surveys of common conditions often wish to measure sexual (dys)function as an explanatory or outcome measure (Boul, 2007; McCabe et al., 2010; Mitchell et al., 2013). Many measures for assessing sexual (dys)function exist, but few are brief, acceptable in nonclinical settings, and relevant to diverse sexual lifestyles (Mitchell, Ploubidis, Datta, & Wellings, 2012). The Natsal-SF is a brief, multidimensional measure that was developed specifically for assessing sexual function in community health surveys of men and women (Mitchell et al., 2012; Mitchell & Wellings, 2013). The 17-item (16 per gender) Natsal-SF was first used to assess sexual function in the British population in the third National Survey of Sexual Attitudes and Lifestyles (Natsal-3) (Mitchell et al., 2013). However, the current scoring method for the Natsal-SF is difficult to calculate without expertise in statistical modeling. This article examines the validity of two simplified methods for scoring the Natsal-SF based on alternate uses of the factor loadings produced from the original model-based sexual function scores. The results from these simplified scoring methods are compared to those from the model-based method, the advantages and disadvantages of the three scoring approaches are discussed, and a flowchart showing how to use the simpler methods is presented.

## Method

Natsal-3 is a national probability sample survey of 15,162 people (57.7% response rate) aged 16 to 74, resident in Britain, undertaken between September 2010 and August 2012. Details of the methodology and question wording are published elsewhere (Erens et al., 2014; Mercer et al., 2013). Briefly, and of relevance to this article, Natsal-3 was administered as a computer-assisted personal interview in participants’ homes, and used computer-assisted self-interview for the more sensitive questions, including those used to derive the Natsal-SF. These questions reflect three components of sexual function: (1) problems with sexual response experienced for three or more months in the past year, (2) sexual function in the relationship context, and (3) participants’ appraisals of their sex lives (Mitchell et al., 2013; Mitchell et al., 2012).

The Natsal-SF scores were originally derived using a general-specific latent variable model in which a global latent factor accounts for the variation in all Natsal-SF items and three specific factors account for the variation in the three components of sexual function (Mitchell et al., 2012). Participants who were sexually active but not in a relationship for all of the past year were not asked the questions on sexual function in the relationship context, so the Full Information Maximum Likelihood method was used to impute their data for these items (Mitchell et al., 2013). They were regarded as having hypothetical relationships; their answers in relation to their hypothetical relationships were assumed to be the same as participants who gave the same responses on other items of the Natsal-SF. The model produced latent sexual function scores for each participant, with a score of approximately zero representing mean sexual function for the British general population. Mitchell and colleagues (2013) inverted the sexual function scores so that a lower score indicated lower sexual function, but here we leave the score in its original format such that higher scores indicate lower sexual function.

We calculated alternative Natsal-SF scores using two simplified scoring methods. Method 1 (M1) uses the factor loadings to three decimal places from the global factor produced by the model-based method to weight each item response for each participant. Items with a Likert format are scored from 0 to 1 with equal intervals between each response option. The factor loading for each item with a Likert-type response is multiplied by the appropriate item score. These scores are then summed to produce a total sexual function score. Method 2 (M2) uses whole numbers to score each item instead of factor loadings. Scores for each component are summed and, if necessary, standardized to represent the weight given to the component in the original model. The scores from each component are then summed to produce a total sexual function score. For both methods, participants without data for the partnership component have their score standardized to the same scale range as scores for participants with full information.

For both M1 and M2, the raw scores were transformed into standard-normal distributions by square rooting and standardizing each score to enable comparisons with the approximately standard-normal distribution of the model-based score. Pearson correlation coefficients for the two raw scores, the standard-normal scores, and the model-based sexual function score were calculated. The distributions were also described with regard to their range, mean, median, variance, skewness, and kurtosis.

The model-based score and the two standard-normal scores were then used in univariate linear regression analysis with key variables examined in a previous article (Mitchell et al., 2013). For simplicity, regression coefficients with overlapping confidence intervals indicate no statistically significant difference between the model-based score and the standard-normal score (M1 and M2). The fewer the differences, the more reliable the standard-normal score. Formal statistical comparisons of these regression coefficients were used to assess the accuracy of using overlapping confidence intervals as markers of significant differences; these data are not shown.

## Results

Of all participants in the Natsal-3 study, 11,472 (75.7%) had been sexually active in the past year (reported at least one sexual partner in the year prior to interview), answered all relevant questions enabling us to derive a Natsal-SF score for them, and were eligible for the analysis presented here. Of these, 4,163 (36.3%) participants were not in a sexual relationship for the whole of the past year and had their relationship component estimated for the model-based score. Table 1 gives the score for each of the 17 items used to derive the Natsal-SF and the maximum possible score for each of the two simpler methods. The Appendix is a flowchart that gives potential users a step-by-step guide to using the two simpler methods.

**Table 1.** Scores for Individual Items Used to Derive the Natsal-SF: A Comparison of Two Simple Scoring Methods

Components and Items	Method 1		Method 2
	Score	Weight	Score
*Sexual problems*	*Max 8*	*Max 3.034*	*Max 14*
1. In the past year, have you experienced any of the following for a period of three months or longer? (Select all that apply)			
Lacked interest in having sex	1	0.472	2
Lacked enjoyment in sex	1	0.500	2
Felt anxious during sex	1	0.441	2
Felt physical pain as a result of sex	1	0.345	2
Felt no excitement or arousal during sex	1	0.479	2
Did not reach a climax (experience an orgasm) or took a long time to reach a climax despite feeling excited/aroused	1	0.301	1
Reached a climax (experienced an orgasm) more quickly than you would like	1	0.130	1
Had an uncomfortably dry vagina/had trouble getting or keeping an erection	1	0.366	2
None of these	0	0.000	0
*Sexual partnership*	*Max 4*	*Max 2.216*	*Max 16 (multiplied by 0.6875)*
2. My partner and I share about the same level of interest in having sex (Select one only)			
Agree strongly	0	0.646	0
Agree	0.25	0.646	1
Neither agree nor disagree	0.5	0.646	2
Disagree	0.75	0.646	3
Disagree strongly	1	0.646	4
3. My partner and I share the same sexual likes and dislikes (Select one only)			
Agree strongly	0	0.573	0
Agree	0.25	0.573	1
Neither agree nor disagree	0.5	0.573	2
Disagree	0.75	0.573	3
Disagree strongly	1	0.573	4
4. My partner has experienced sexual difficulties in the past year (Select one only)			
Agree strongly	1	0.468	4
Agree	0.75	0.468	3
Neither agree nor disagree	0.5	0.468	2
Disagree	0.25	0.468	1
Disagree strongly	0	0.468	0
5. I feel emotionally close to my partner when we have sex together (Select one only)			
Always	0	0.529	0
Most of the time	0.25	0.529	1
Sometimes	0.5	0.529	2
Not very often	0.75	0.529	3
Hardly ever	1	0.529	4
*Overall sex life*	*Max 4*	*Max 2.580*	*Max 13*
6. I feel satisfied with my sex life (Select one only)			
Agree strongly	0	0.838	0
Agree	0.25	0.838	1
Neither agree nor disagree	0.5	0.838	2
Disagree	0.75	0.838	3
Disagree strongly	1	0.838	4
7. I feel distressed or worried about my sex life (Select one only)			
Agree strongly	1	0.799	4
Agree	0.75	0.799	3
Neither agree nor disagree	0.5	0.799	2
Disagree	0.25	0.799	1
Disagree strongly	0	0.799	0
8. I have avoided sex because of sexual difficulties, either my own or those of my partner (Select one only)			
Agree strongly	1	0.702	4
Agree	0.75	0.702	3
Neither agree nor disagree	0.5	0.702	2
Disagree	0.25	0.702	1
Disagree strongly	0	0.702	0
9. Have you sought help or advice regarding your sex life from any of the following sources in the past year? (Select one only)			
None	0	0.241	0
At least one of the listed sources	1	0.241	1
*Total possible score (for participants reporting no sexual relationship for whole of past year)*	12	5.614	27
*Total possible score (for participants reporting sexual relationship for whole of past year)*	16	7.830	38

**Figure 1:**
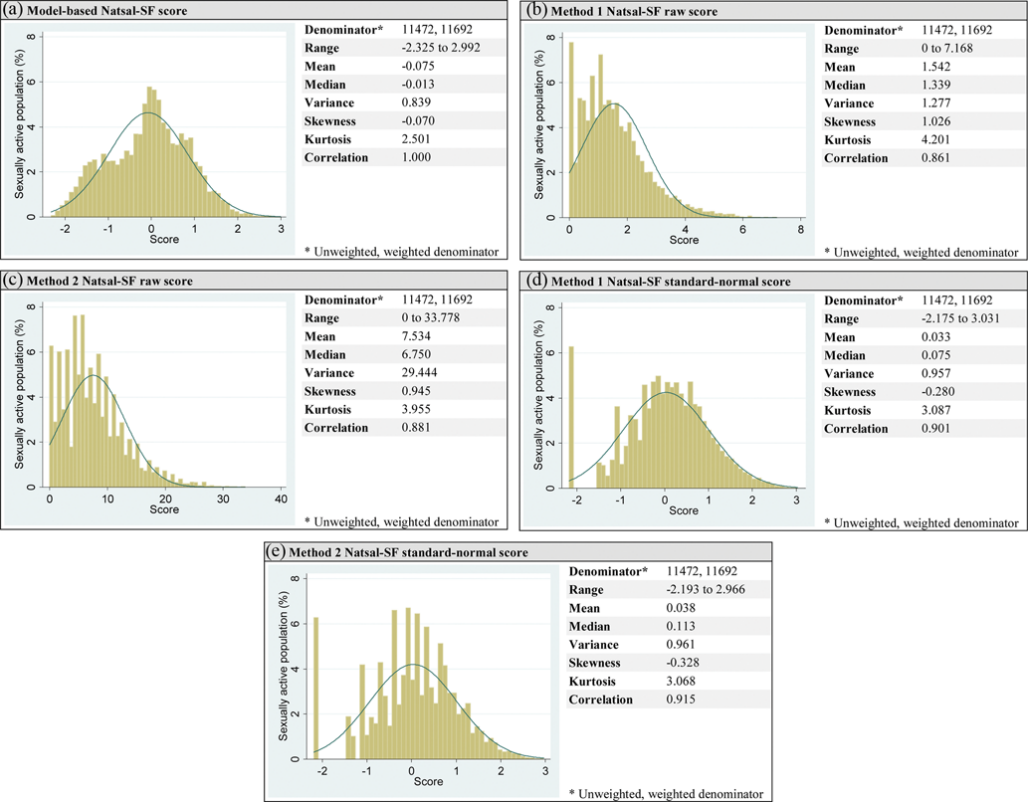
Distributions and summary statistics for model-based score and Method 1 and Method 2 (raw and standard-normal scores).

The scores for M1 and M2 correlated highly with the model-based score (raw 0.8613; standard-normal 0.9013 for M1, raw 0.8813; standard-normal 0.9145 for M2); see Figure 1 (a), (b), (c), (d). The raw distributions for M1 and M2 are not directly comparable to the model-based distribution—refer to Figure 1 (b),(c)—and so the standard-normal distributions were used for comparison. These distributions closely resemble the model-based distribution, despite being more negatively skewed and more peaked; see Figure 1 (a), (d), (e). The gap between the minimum score and the next score and the peak that these zero scores generate accounts for the difference in skewness and kurtosis, respectively. The standard-normal distribution for M1 better matches the model-based distribution in mean, median, variance, and skewness, while the distribution for M2 better matches the model-based distribution in range and kurtosis.

The regression coefficients produced with the model-based score were similar to the coefficients produced using the standard-normal scores for M1 and M2 across the previously identified key variables (Table 2). However, the scores performed differently across gender and relationship context. M1 and M2 suggest that women have significantly higher sexual function *scores* than men (where higher scores indicate lower sexual function), while the model-based score shows that women have higher sexual function scores, but that the difference is not significant. The coefficients for women for M1 and M2 are significantly different from the coefficient for the model-based score. The model-based score coefficients for those who are not currently in a relationship indicate that they have higher sexual function scores than those who are living together or in a noncohabiting relationship. In contrast, the coefficients produced by M1 and M2 suggest that there is no difference between those not in a relationship and those who are living together. The coefficients for M1 and M2 for those not in a relationship are significantly different from the model-based coefficients. The differences in all regression coefficients are supported by formal statistical comparisons, both within and across methods.

**Table 2.** Natsal-SF Comparison of Correlation Coefficients From Using a Model-Based Approach to Two Simple Scoring Methods

Variable	Model Based		Method 1*		Method 2*	
	Coefficient	95% CI	Coefficient	95% CI	Coefficient	95% CI
Respondent's sex						
Male	base	—	base	—	base	—
Female	0.025	[−0.013;0.064]	0.146	[0.106;0.186]	0.123	[0.082;0.163]
Age group						
16–24	base	—	base	—	base	—
25–34	0.085	[0.037;0.133]	0.153	[0.100;0.206]	0.153	[0.099;0.206]
35–44	0.133	[0.076;0.190]	0.184	[0.123;0.244]	0.185	[0.125;0.245]
45–54	0.195	[0.133;0.256]	0.226	[0.159;0.294]	0.239	[0.172;0.307]
55–64	0.379	[0.313;0.446]	0.398	[0.327;0.469]	0.421	[0.350;0.492]
65–74	0.387	[0.311;0.463]	0.343	[0.265;0.420]	0.382	[0.304;0.460]
Quintiles of Index of Multiple Deprivation						
1 (least deprived)	base	—	base	—	base	—
2	−0.022	[−0.084;0.041]	−0.033	[−0.098;0.033]	−0.033	[−0.099;0.033]
3	−0.036	[−0.103;0.032]	−0.059	[−0.130;0.012]	−0.059	[−0.130;0.012]
4	−0.026	[−0.090;0.037]	−0.054	[−0.119;0.011]	−0.053	[−0.119;0.012]
5 (most deprived)	0.030	[−0.032;0.092]	−0.040	[−0.104;0.024]	−0.033	[−0.098;0.031]
Self-reported health						
Very good/good	base	—	base	—	base	—
Fair	0.338	[0.277;0.399]	0.330	[0.267;0.393]	0.341	[0.278;0.404]
Bad/very bad	0.504	[0.383;0.626]	0.499	[0.372;0.626]	0.507	[0.380;0.634]
Current depression (PHQ-2)						
No	base	—	base	—	base	—
Yes	0.631	[0.567;0.694]	0.683	[0.617;0.748]	0.680	[0.615;0.744]
Relationship context						
Living together	base	—	base	—	base	—
In a noncohabiting relationship	−0.240	[−0.289; −0.191]	−0.292	[−0.345; −0.239]	−0.300	[−0.353; −0.246]
Previously in a relationship but not now	0.272	[0.212;0.333]	0.052	[−0.016;0.120]	0.056	[−0.012;0.124]
Never married/lived with someone	0.158	[0.105;0.212]	−0.036	[−0.097;0.024]	−0.038	[−0.099;0.023]
Happy in current relationship						
Yes	base	—	base	—	base	—
Else	0.680	[0.634;0.727]	0.625	[0.576;0.674]	0.625	[0.576;0.675]
Four or more sex acts in the past four weeks						
Yes	base	—	base	—	base	—
No	0.551	[0.512;0.590]	0.503	[0.462;0.544]	0.515	[0.473;0.556]
STI diagnoses (excluding thrush), past five years						
No	base	—	base	—	base	—
Yes	0.072	[−0.009;0.154]	0.095	[0.001;0.189]	0.084	[−0.009;0.177]

*Standard-normal score used for linear regression model.

## Discussion

The simplified scoring methods described here enable researchers without statistical modeling expertise or specialized software to use the Natsal-SF—a novel, validated, multidimensional measure of sexual function designed for community surveys (Mitchell et al., 2012; Mitchell et al., 2013). The two methods result in distributions similar to that of the original model-based sexual function score, correlate highly with the original score, and have similar relationships with previously identified key variables as observed for the original score (Mitchell et al., 2013). M2 performs slightly better than M1, is simpler to calculate, and so is the preferred method for use in future research. However, the scores generated from M1 and M2 differ from the model-based score in two ways.

While statistically the distributions of the simplified scores are similar to the distribution of the model-based score, the visual differences are more pronounced. Participants with no sexual function problems appear around the lower-bound tail of the model-based distribution because of the influence of other variables and covariance terms, but score zero in the simplified distributions resulting in a peak of zero scores and a gap between the next score. This explains the difference in both kurtosis and skewness between the simplified distributions and the model-based distribution.

The simplified scores also differ from the model-based score in gender and relationship context. However, the gender differences are not large, and the coefficients are in the same direction as the model-based score; because analysis is conventionally split by gender, these differences are unlikely to be important. The difference in relationship context is almost entirely due to the model-based method using imputed data for participants who were not in a relationship during the past year, while the simplified methods standardized scores without partnership data to the same scale as those with partnership data. This will unlikely be fixed without complex methods of data imputation, but in subsequent analysis may be lessened by controlling for relationship context.

The Natsal-SF was originally designed to measure and study the distribution of sexual function in the British population. The alternate scoring methods simplify deriving a sexual function score to increase the utility of the Natsal-SF measure. However, the factor loadings used to derive the simplified scores are specific to the British population and may differ in other populations and cultural contexts. There may also be issues translating the Natsal-SF items, which could result in a differently weighted model. As the simplified scoring methods are based on factor loadings derived from the Natsal-3 sample, they may be less reliable in these settings. Consequently, studies using the Natsal-SF would ideally model the score using a general-specific model, as we originally did (Mitchell et al., 2013), to accurately understand sexual function in their study population. However, this may not be feasible for smaller study populations, as complex statistical modeling typically requires large samples to produce representative population scores.

Standard-normal scores for the two simplified methods were used in these analyses for *scaled* comparison with the model-based score. However, raw scores would further ease interpretation, with a clear zero score where higher scores indicate poorer sexual function, and should be used instead of standard-normal scores. Finally, we do not recommend individual assessment using these simplified scoring methods as the resulting score would have no clinical relevance or meaning. The score can be considered relative only to sexual function in a study population.

Future studies that use a general-specific model with the Natsal-SF measure will each produce their own set of factor loadings. These loadings may vary across populations but are likely to produce similar patterns to the Natsal-3 population. Such research would further validate using the simplified scoring methods across different populations and further increase the utility of the Natsal-SF in epidemiological research. In the meantime, we hope that the flowchart presented in the Appendix will be a useful resource for researchers wishing to employ either of the simplified Natsal-SF measures.
